# A Simple Measure to Assess Hyperinflation and Air Trapping: 1-Forced Expiratory Volume in Three Second / Forced Vital Capacity

**DOI:** 10.4274/balkanmedj.2015.0857

**Published:** 2017-03-28

**Authors:** Sermin Börekçi, Tunçalp Demir, Aslı Görek Dilektaşlı, Melahat Uygun, Nurhayat Yıldırım

**Affiliations:** 1 Department of Pulmonology, İstanbul University Cerrahpaşa School of Medicine, İstanbul, Turkey; 2 Department of Pulmonology, Uludag University School of Medicine, Bursa, Turkey

**Keywords:** 1-FEV3/FVC, hyperinflation, spirometry

## Abstract

**Background:**

Several recent studies have suggested that 1 minus-forced expiratory volume expired in 3 seconds / forced vital capacity (1-FEV_3_/FVC) may be an indicator of distal airway obstruction and a promising measure to evaluate small airways dysfunction.

**Aims:**

To investigate the associations of 1-FEV_3_/FVC with the spirometric measures and lung volumes that assess small airways dysfunction and reflects hyperinflation and air trapping.

**Study Design:**

Retrospective cross-sectional study.

**Methods:**

Retrospective assessment of a total of 1110 cases who underwent body plethysmographic lung volume estimations between a time span from 2005 to 2012. Patients were assigned into two groups: firstly by FEV_1_/FVC (FEV_1_/FVC <70% vs. FEV_1_/FVC ≥70%); secondly by FEV_3_/FVC < lower limits of normal (LLN) (FEV_3_/FVC < LLN vs. FEV_3_/FVC ≥ LLN). Spirometric indices and lung volumes measured by whole-body plethysmography were compared in groups. Also the correlation of spirometric indices with measured lung volumes were assessed in the whole-study population and in subgroups stratified according to FEV_1_/FVC and FEV_3_/FVC.

**Results:**

Six hundred seven (54.7%) were male and 503 (45.3%) were female, with a mean age of 52.5±15.6 years. Mean FEV_3_/FVC and 1-FEV_3_/FVC were 87.05%, 12.95%, respectively. The mean 1-FEV_3_/FVC was 4.9% in the FEV_1_/FVC ≥70% group (n=644) vs. 24.1% in the FEV_1_/FVC <70% group (n=466). A positive correlation was found between 1-FEV_3_/FVC and residual volume (r=0.70; p<0.0001), functional residual capacity-pleth (r=0.61; p<0.0001), and total lung capacity (r=0.47; p<0.0001). 1-FEV_3_/FVC was negatively correlated with forced expiratory flow2_5-75_ (r=−0.84; p<0.0001). The upper limit of 95% confidence interval for 1-FEV_3_/FVC was 13.7%. 1-FEV_3_/FVC showed significant correlations with parameters of air trapping and hyperinflation measured by whole-body plethysmography. Importantly, these correlations were higher in study participants with FEV_1_/FVC <70% or FEV_3_/FVC < LLN compared to those with FEV_1_/FVC ≥70% or FEV_3_/FVC ≥ LLN, respectively.

**Conclusion:**

1-FEV_3_/FVC can be easily calculated from routine spirometric measurements. 1-FEV_3_/FVC is a promising marker of air trapping and hyperinflation. We suggest that 1-FEV_3_/FVC is complementary to FEV_1_/FVC and recommend clinicians to routinely report and evaluate together with FEV_1_/FVC during spirometry.

The ratio of the forced vital capacity (FVC) that is not yet expired within the first 3 seconds of a forced exhalation is expressed with the following formula: 1 minus-forced expiratory volume in third seconds (1-FEV_3_) / FVC (1,2). Originally, Hansen et al. (3) showed that 1-FEV_3_/FVC may be used for the evaluation of small airways, may be an indicator of the distal expiratory obstruction and was more sensitive than forced expiratory flow (FEF)_25-75_ % in evaluating small airways (3).

In chronic obstructive pulmonary diseases (COPD), although small airways are mainly involved, larger airways are also affected due to a number of factors, including the loss of ciliated epithelial cells, squamous metaplasia, thickening of the basement membrane, mucous gland hypertrophy and hyperplasia (4). All these factors contribute to irreversible obstruction mainly caused by progressive air trapping, which is a prominent feature of COPD. Both the peripheral and proximal airways are also affected not only in COPD but also in asthma.

The forced expiratory volume in the first second (FEV_1_) mainly reflects large airways obstruction, and for FEV_1_ to become abnormal a significant amount of small airways must be affected (5). Later fractions of forced exhalation those occur after FEV_1_, such as FEV_3_ was proposed to be more sensitive to reductions in terminal expiratory flow (1,3). For that reason, FEV_3_, FEV_3_/FVC ratio and 1-FEV_3_/FVC were suggested to better assess small airways disease (3,6,7,8). Therefore, both in asthma and COPD, 1-FEV_3_/FVC may be an indicator of small airways dysfunction and air trapping.

In order to detect the presence of air trapping in the lungs, lung volumes should be measured to determine the total lung capacity and the residual volume. However, since these methods are associated with increased medical costs and require sophisticated equipment, they are not widely utilized. However, 1-FEV_3_/FVC value can be readily calculated by the widely available standard spirometric examination, and thus may help to detect air trapping in patients with obstructive pulmonary disease. In order to test this hypothesis, the present study aimed to investigate the associations of 1-FEV_3_/FVC in obstructive lung diseases and its relationship with the spirometric measures and lung volumes that assess small airways dysfunction, which reflects hyperinflation and air trapping.

## MATERIALS AND METHODS

A retrospective assessment of a total of 1110 participants with at least three acceptable spirometric manoeuvres who underwent body plethysmographic lung volume estimations (ZAN 500 Plethysmography, nSpire, Germany) between 2005 and 2012 at the Pulmonary Function Test Laboratory was carried out. Repeated tests of same person were excluded (according to duplicated name, surnames and identity card numbers). None of the authors have reported a conflict of interest prior to the study. The pulmonologists reviewed all of the pulmonary function tests on a daily basis. The technicians were trained in whole-body plethysmography techniques, and the laboratory supervisor also checked all the steps involved in the test procedures in terms of adherence to the American Thoracic Society and American Thoracic Society/European Respiratory Society guidelines (9,10,11,12). The whole-body plethysmography device was calibrated daily according to manufacturer’s guidelines and biological quality control was performed on a monthly basis.

Patients younger than 18 years of age were excluded, and only pre-bronchodilator test results were utilized. 1-FEV_3_/FVC was calculated electronically by whole-body plethysmography for each patient; this can also be calculated by spirometers.

1-FEV_3_/FVC estimation: After FEV_3_ and FEV_3_/FVC measurements were obtained from records of the patients, 1-FEV_3_/FVC was calculated to show the remaining unexhaled vital capacity ratio in the lung at the end of the 3^rd^ second [(FVC-FEV_3_)/FVC=1-FEV_3_/FVC].

There is controversy regarding appropriate criteria to define airflow obstruction by using the fixed threshold of 70% or the lower limits of normal (LLN) for the FEV_1_/FVC ratio (13). In the present study, firstly, we defined airflow obstruction by using the fixed threshold of 70% for the FEV_1_/FVC ratio by using pre-bronchodilator spirometry (14,15). Patients were assigned into either the group with FEV_1_/FVC <70% or the group with FEV_1_/FVC ≥70%. The two groups were compared in terms of FVC, FEV_1_, FEV_1_/FVC, FEF_25-75_, inspiratory capacity (IC), total lung capacity (TLC), residual volume (RV), RV/TLC, thoracic gas volume at functional residual capacity (FRC-pleth), FEV_3_, FEV_3_/FVC, and 1-FEV_3_/FVC.

Secondly, in order to assess whether FEV_3_/FVC (accordingly, 1-FEV_3_/FVC) provides additional information on air trapping and hyperinflation to that of FEV_1_/FVC, we analysed correlations of FEV_3_/FVC abnormality. We defined FEV_3_/FVC abnormality by using the redefined LLN criteria for FEV_3_/FVC (16). Analyses were performed separately, for the whole study population, and the subgroups, including individuals with FEV_3_/FVC < LLN and FEV_3_/FVC ≥ LLN.

### Statistical analyses

Statistical analyses were performed using Statistical Package for Social Sciences (SPSS) software version 21.0 (IBM SPSS Statistics for Windows, Armonk, NY: IBM Corp.) Continuous variables were expressed as mean ± standard deviation, whereas categorical variables were shown as the number and percentage of cases. Means and medians were compared using Student’s t-test or Mann-Whitney U-test, depending on the normality distribution of data. A p value <0.05 was considered an indication of statistical significance. In addition, the correlations between variables were tested using Spearman’s correlation analysis. The study protocol was approved by the Ethics Board (Approval No: 83045809/604.01/02-346067).

## RESULTS

Of the overall study population, 607 (54.7%) were male, and 503 (45.3%) were female, with a mean age of 52.5±15.6 years, mean FEV_3_/FVC of 87.05% and 1-FEV_3_/FVC of 12% (Table 1).

Of the total study population, 644 had a FEV_1_/FVC ratio ≥70%, and 466 had FEV_1_/FVC <70%. Mean FEV_3_/FVC was 95.1% in the group with FEV_1_/FVC ≥70% and 75.9% in the group with FEV_1_/FVC <70%, while the corresponding 1-FEV_3_/FVC values in these two groups were 4.9% and 24.1%, respectively (Table 2). The upper 95% confidence limit for 1-FEV_3_/FVC was 13.7%.

Individuals with FEV_1_/FVC <70% had a significantly higher TLC, RV, FRCpleth, RV/TLC, and a significantly more reduced IC than those with FEV_1_/FVC ≥70% (Table 2). 1-FEV_3_/FVC had moderate to strong and significant correlations with RV (r=0.70; p<0.0001), FEF_25-75_ (r=−0.84; p<0.0001), RV/TLC (r=0.59; p<0.0001), TLC (r=0.47; p<0.0001) and FRCpleth (r=0.61; p<0.0001) in the total study population (Table 3). When analysed separately in the FEV_1_/FVC ≥70% and FEV_1_/FVC <70% groups, we observed that 1-FEV_3_/FVC had significant correlations with RV, RV/TLC, TLC, FRC pleth and FEF_25-75_ (Table 3). Importantly, 1-FEV_3_/FVC displayed stronger correlations with RV, RV/LC, TLC, FRCpleth and FEF_25-75_ in those with FEV_1_/FVC <70% compared to those with FEV_1_/FVC ≥70% (Table 3). On the other hand, correlation of 1-FEV_3_/FVC with IC was weak in the total study population and both FEV_1_/FVC ≥70% and FEV_1_/FVC <70% groups (Table 3).

In a further analysis, we assessed FEV_3_/FVC normality by the newly defined FEV_3_/FVC LLN criteria. A total of 379 (34.1%) of the whole study population were below the LLN for FEV_3_/FVC. Individuals with FEV_3_/FVC < LLN had a significantly higher TLC, RV and RV/TLC, and a significantly more reduced IC than those in the group with FEV_3_/FVC ≥ LLN (Table 4). 1-FEV_3_/FVC had significant correlations with RV, RV/TLC, TLC, FRCpleth and FEF_25-75_ in both FEV_3_/FVC ≥ LLN and FEV_3_/FVC < LLN groups. 1-FEV_3_/FVC displayed stronger correlations with RV, RV/TLC, TLC, FRCpleth and FEF_25-75_ in those with FEV_3_/FVC < LLN compared to those with FEV_3_/FVC ≥ LLN (Table 4). More importantly, we observed somewhat higher correlation coefficients for FEV_3_/FVC with IC, FEF_25-75_ and the air trapping measures - RV and RV/TLC - in FEV_3_/FVC < LLN subgroup than the correlations observed in FEV_1_/FVC <70% subgroup (Table 5).

We also observed that FEV_1_/FVC has a similar or slightly higher level of correlation with TLC, RV, FRCpleth, RV/TLC and FEF_25-75_ in the total study population and subgroup analyses (Table 3). But when airflow obstruction is defined by FEV_3_/FVC LLN criterion instead of FEV_1_/FVC, we observed that 1-FEV_3_/FVC displays a stronger correlation with TLC (r=0.66, p<0.0001), RV (r=0.67, p<0.0001), RV/TLC (r=0.55, p<0.0001), FRCpleth (r=0.66, p<0.0001), FEF_25-75_ (r=-0.82, p<0.0001) and even with IC (r=0.30, p<0.0001) (Table 4).

## DISCUSSION

In the present study, we report that the fraction of FVC that has not been expired at the end of the first three seconds of the FVC (1-FEV_3_/FVC), is significantly increased in patients with a FEV_1_/FVC below 70%. Both groups, including FEV_1_/FVC <70% and FEV_3_/FVC < LLN subjects, had significantly increased hyperinflation and air trapping with regard to RV, RV/TLC, TLC compared to FEV_1_/FVC ≥70% and FEV_3_/FVC ≥ LLN groups, respectively. We also showed that 1-FEV_3_/FVC significantly correlates with measures of hyperinflation and air trapping in the whole study population as well as in subgroup analyses, including FEV_1_/FVC <70% and FEV_3_/FVC < LLN subjects.

Small airways are major contributors to airflow limitation in asthma and COPD (17). Air trapping and premature airway closing are accepted as useful surrogates to assess and quantify small airways obstruction. RV and RV/TLC ratios are useful and widely accepted measures of hyperinflation and air trapping (18).

The earliest change associated with airflow obstruction is a reduction in the terminal portion of the spirogram, even though the initial part of the spirogram is barely affected (9). In this context, later fractions of forced exhalation, i.e. those that occur after the first second of exhalation, such as FEV_3_, were proposed to define reductions in terminal expiratory flow (1,3). FEV_3_ and FEV_3_/FVC were introduced in the last three decades, first by Crapo et al. (19) in 1981, followed by Miller et al. (20,21) in 1985. Later on, Hansen et al. (16) introduced the concept of 1-FEV3/FVC to identify the increased fraction of the long-time-constant lung units as a measure of late expiratory fraction in their study utilizing data from a smokers and never-smokers population of the Third National Health and Nutrition Examination Survey (22). Our study shows that 1-FEV_3_/FVC is a promising spirometric parameter that correlates with markers of air trapping and hyperinflation. 1-FEV_3_/FVC can be easily calculated by using standard spirometry through the measurement of FEV_3_ at the 3^rd^ second of the forced expiratory manoeuvre. We suggest that 1-FEV_3_/FVC may be used to assess the presence of hyperinflation and air trapping, especially in settings where the lung volumes cannot be measured. Furthermore, FEV_3_/FVC LLN criteria define a group with significantly worse spirometric indices (FEV_1_, FEV_3_, FEV_1_/FVC, FEF_25-75_), and increased RV, RV/TLC, TLC compared to FEV_3_/FVC ≥ LLN subjects.

Previously, FEV_3_/FVC and 1-FEV_3_/FVC were reported to be superior to FEF_25-75_ in the assessment of expiratory airflow limitation, since FEF_25-75_ can be misleading, with a high rate of false-negative and false-positive results (3,22). We observed that FEF_25-75_ had a high correlation with 1-FEV_3_/FVC in the total study population as well as in subgroup analyses. Interestingly, we found that FEF_25-75_ had a higher correlation with RV/TLC and IC than that of 1-FEV3/FVC, whereas 1-FEV_3_/FVC had a higher correlation with RV, TLC and FRCpleth than that of FEF_25-75_. But as we did not define normality vs. abnormality according to LLN for FEF_25-75_, our analysis did not allow a comparison of our results with previous findings.

In addition to these results, we also observed that not only FEV_3_/FVC but also FEV_1_/FVC was negatively correlated with RV (r=−0.75; p<0.001), RV/TLC (r=−0.63; p<0.001) and TLC (r=0.49; p<0.001). We think this finding is consistent with Hansen’s suggestion that FEV_1_/FVC and FEV_3_/FVC are complementary and both ratios are beneficial in the characterization of expiratory airflow obstruction (3). Current studies and our own are still unable to answer the question of which ratio is better, 1-FEV_3_/FVC or the FEV_1_/FVC, in diagnosing expiratory airflow obstruction.

The potential strengths of this study include the fact that the pulmonary function test laboratory where all of the tests were performed is the most comprehensive and qualified laboratory in the country, accepting referrals for whole-body plethysmography from more than 40 hospitals. For that reason, we believe our analysis reflects a wide range of a patient profile based on reliable measurements. However, its retrospective design with a lack of detailed history of smoking and other exposures, limited us in investigating the effect of smoking on spirometric measures effects of smoking on spirometric measures. In addition, our database does not include the necessary information regarding the medication history of the study participants. This was another limitation of our study. Nevertheless, whether the FEV_3_/FVC ratio translates into clinically meaningful disease-centred outcomes needs to be evaluated in further observations, together with clinical and radiologic features.

## CONCLUSION

1-FEV_3_/FVC can be easily calculated from routine daily spirometric measurements. 1-FEV_3_/FVC is a promising marker of air trapping and hyperinflation. We suggest that 1-FEV_3_/FVC is complementary to FEV_1_/FVC and recommend clinicians routinely report this measurement and evaluate it together with FEV_1_/FVC during spirometry.

## Figures and Tables

**Table 1:**
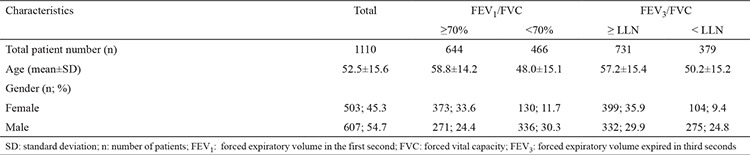
Characteristic features of the study participants

**Table 2:**
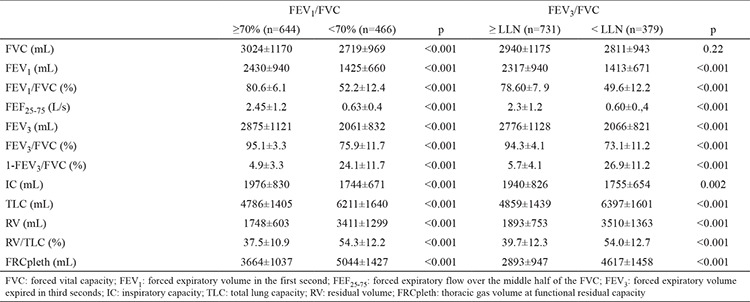
Comparison of spirometric measures and lung volumes between groups with FEV_1_/FVC <70% (n=466) vs. FEV_1_/FVC ≥70 (n=644) and FEV_3_/FVC < LLN (n=379) vs. FEV_3_/FVC ≥ LLN (n=731)

**Table 3:**
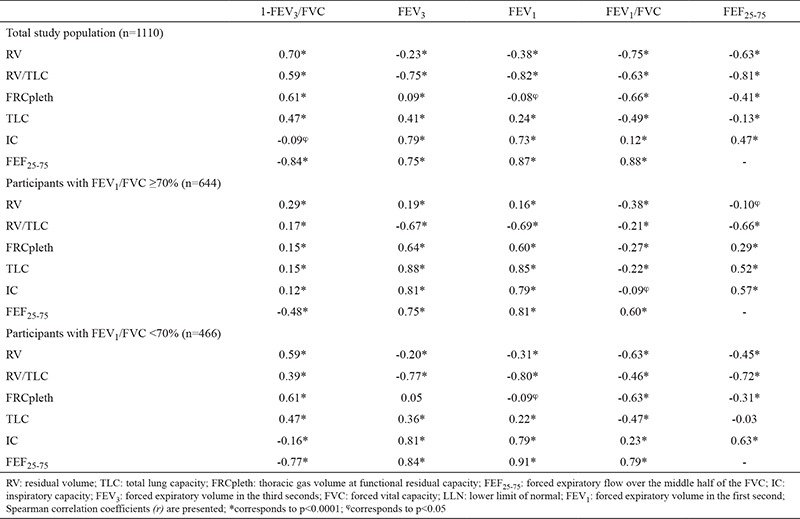
Correlation matrix of 1-FEV_3_/FVC, FEV_1_ and FEV_1_/FVC with RV, FRCpleth, TLC and FEF_25-75_ in the whole study population, and subgroups with FEV_1_/FVC ≥70% and FEV_1_/FVC <70%

**Table 4:**
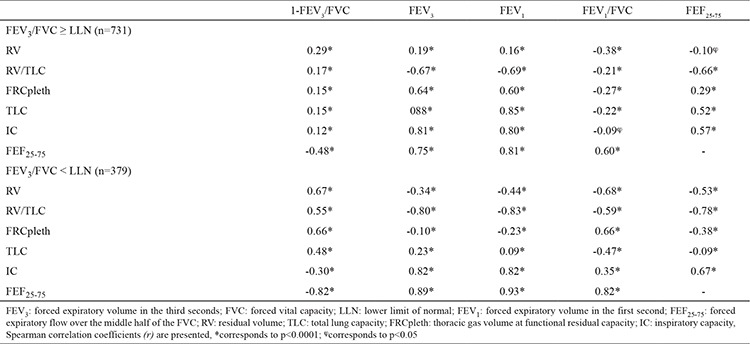
Correlation matrix of 1-FEV_3_/FVC, FEV_1_ and FEV_1_/FVC with RV, FRCpleth, TLC and FEF_25-75_ in FEV_3_/FVC ≥ LLN (n=731) and FEV_3_/FVC < LLN (n=379) subgroups

**Table 5:**
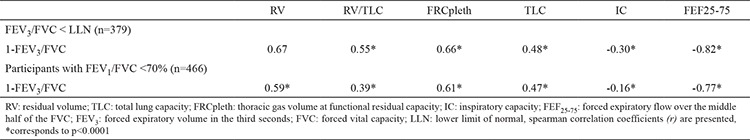
Comparison of correlation coefficients of 1-FEV_3_/FVC with RV, TLC and FEF_25-75_ in FEV_3_/FVC < LLN (n=379) and FEV_1_/FVC <0.70 (n=466) subgroups
